# Human Coronavirus Infection Reorganizes Spatial Genomic Architecture in Permissive Lung Cells

**DOI:** 10.21203/rs.3.rs-3979539/v1

**Published:** 2024-03-13

**Authors:** Ankush Singhal, Cullen Roth, Sofiya N. Micheva-Viteva, Vrinda Venu, Anna Lappala, Jeannie T. Lee, Shawn R. Starkenburg, Christina R. Steadman, Karissa Y. Sanbonmatsu

**Affiliations:** 1 Theoretical Biology and Biophysics, Los Alamos National Laboratory, Los Alamos,NM, USA.; 2 Genomics and Bioanalytics, Los Alamos National Laboratory, Los Alamos, NM, USA.; 3 Biochemistry and Biotechnology, Los Alamos National Laboratory, Los Alamos, NM, USA.; 4 Climate, Ecology & Environment, Los Alamos National Laboratory, Los Alamos, NM, USA.; 5 Department of Genetics, The Blavatnik Institute, Harvard Medical School, Boston, USA; 6 Departement of Molecular Biology, Massachusetts General Hospital, Boston, USA

## Abstract

Chromatin conformation capture followed by next-generation sequencing in combination with large-scale polymer simulations (4DHiC) produces detailed information on genomic loci interactions, allowing for the interrogation of 3D spatial genomic structures. Here, Hi-C data was acquired from the infection of fetal lung fibroblast (MRC5) cells with *α*-coronavirus 229E (CoV229E). Experimental Hi-C contact maps were used to determine viral-induced changes in genomic architecture over a 48-hour time period following viral infection, revealing substantial alterations in contacts within chromosomes and in contacts between different chromosomes. To gain further structural insight and quantify the underlying changes, we applied the 4DHiC polymer simulation method to reconstruct the 3D genomic structures and dynamics corresponding to the Hi-C maps. The models successfully reproduced experimental Hi-C data, including the changes in contacts induced by viral infection. Our 3D spatial simulations uncovered widespread chromatin restructuring, including increased chromosome compactness and A-B compartment mixing arising from infection. Our model also suggests increased spatial accessibility to regions containing interferon-stimulated genes upon infection with CoV229E, followed by chromatin restructuring at later time points, potentially inducing the migration of chromatin into more compact regions. This is consistent with previously observed suppression of gene expression. Our spatial genomics study provides a mechanistic structural basis for changes in chromosome architecture induced by coronavirus infection in lung cells.

## Introduction

Many vital processes, such as DNA transcription, replication, and repair [1, 2], depend upon the structural organization of chromosomes. This organization is characterized by folding into different structural motifs from chromosome territories, transcriptional compartments [3], topologically associated domains (TADs) [4, 5], and loops [6]. Significant progress has been made in understanding the molecular mechanisms of three-dimensional chromosome structure, including the requirement of loop extrusion and compartmentalization of chromatin domains and loops [7, 8, 9]. However, mechanisms governing chromosome localization, volume, and shape still remain poorly understood. Disruption in chromosome structure underlying structural changes in chromosome compartments are correlated with changes in contacts among genes, potentially leading to altered expression. It has been shown that viral species may disrupt host chromatin architecture, thereby neutralizing the host immune response and potentially result- ing in long-term effects [10, 11]. Human coronavirus (HCoV) infections are known to cause mild upper-respiratory tract illness. In lung cancer cells with high levels of ACE2, severe acute respiratory syndrome coronavirus 2 (SARS- CoV-2) restructures chromatin with cohesin depletion from intra-TAD regions [12]. Given the role of cohesin protein complexes in supporting chromosome structural maintenance and DNA loop extrusion, depletion results in intra-TAD weakening [12]. This weakening likely benefits the SARS-CoV-2 infection by dysregulation of the antiviral response. These changes are thought to be unique to SARS-CoV-2, given that other coronaviruses (HCoV-OC43) and synthetic RNA viral mimics do not produce similar chromatin changes [12]. However, the impact of coronaviruses on the spatial genome architecture of more relevant, non-cancerous cells, such as fetal lung fibroblast cells (MRC5), is still not well understood, nor is the impact of less pathogenic viruses such as Cov229E.

To ascertain the effect of viruses on genome structure, chromatin conformation capture-based assays combined with next-generation sequencing (Hi-C) are applied for measuring the frequency and locations of chromatin contacts [13]. This data produces two-dimensional contact matrices that, while informative, lack 3D spatial information, which allows for the identification of rare long-range contacts [14, 15]. These long-range contacts may be important for genomic function, but the relationship between structure and function is not well characterized. To account for this lack of information, various imaging modalities have also been used to study genome organization [16, 17, 18]. They have shown that the nuclear localization of chromosomes is nonrandom in nature, and gene-rich regions have particular locations within the nucleus [19]. While imaging techniques are ideal for spatial chromosome studies, they are limited by low throughput and require extensive data sets for statistical relevance. A more comprehensive picture of genome organization can be created by integrating these experimental techniques with polymer simulation. Further, modeling methodologies that are experimentally informed may provide greater insight into genome structural dynamics, particularly in response to pathogens [20].

Previously, techniques were developed to model chromosomes at different resolutions as polymer models. Some studies have incorporated one-dimensional information derived from experiments to generate 2D connectivity profiles for chromosomes [21, 22]. In contrast, others have generated structures by minimizing energy functions for the polymer model [23, 24]. Each has provided significant insight into various biological phenomena such as proteinmediated loop formation [25, 26, 27, 28], phase separation and compartmentalization [22, 8]. Similarly, chromosome structures have been successfully recapitulated by applying Hi-C data to identify structural features of interphase and metaphase chromosomes [29]. We have also previously developed the 4DHiC pipeline, that extracts 2D information from Hi-C maps and implements harmonic constraints obtained directly from the Hi-C map onto polymer simulations to reconstruct the 3D organization of the chromosomes. This was applied to the X chromosome while transitioning from an active to inactive state [20]. Here, we extend the capability of this method and apply it to different chromosomes in response to viral infection. This strategy also leads to relevant simulated genome structure, as all the model parameters are derived from the experimental Hi-C data. Our method also scales with experimental data; that is, the resolution of the Hi-C map determines the number of particles in the model. Integrating experimental Hi-C data and in-silico techniques is an effective strategy for modeling 3D genome organization and studying the structural changes that occur over time [20, 30, 24].

Here, we performed Hi-C experiments on fetal lung fibroblast (MRC5) cells infected with *α*-coronavirus 229E (CoV229E). Using the 4DHiC polymer modeling pipeline, we reconstructed 3D chromatin dynamics over a 48-hour time period following viral infection and identified significant alterations in spatial genomic structure during the late stages of infection. We observed notable structural differences in the global chromosome organization, including A and B compartmentalization and increased chromosome compactness, arising from infection. Structural changes were also detected in chromatin regions containing genes involved in pro-inflammatory and interferon responses. We find that regions containing interferon-stimulated genes display increased spatial accessibility upon infection with CoV229E. This is followed by chromatin restructuring at later time points, potentially inducing the migration of chromatin into more compact regions, in line with previously observed suppression of gene expression. Overall, our simulations support experimental evidence that less pathogenic viruses, such as the common cold, can impact chromatin dynamics and structure in host cells.

## Results

### Coronavirus 229E induces global restructuring of chromatin architecture in permissive human lung cells

**Figure 1:**
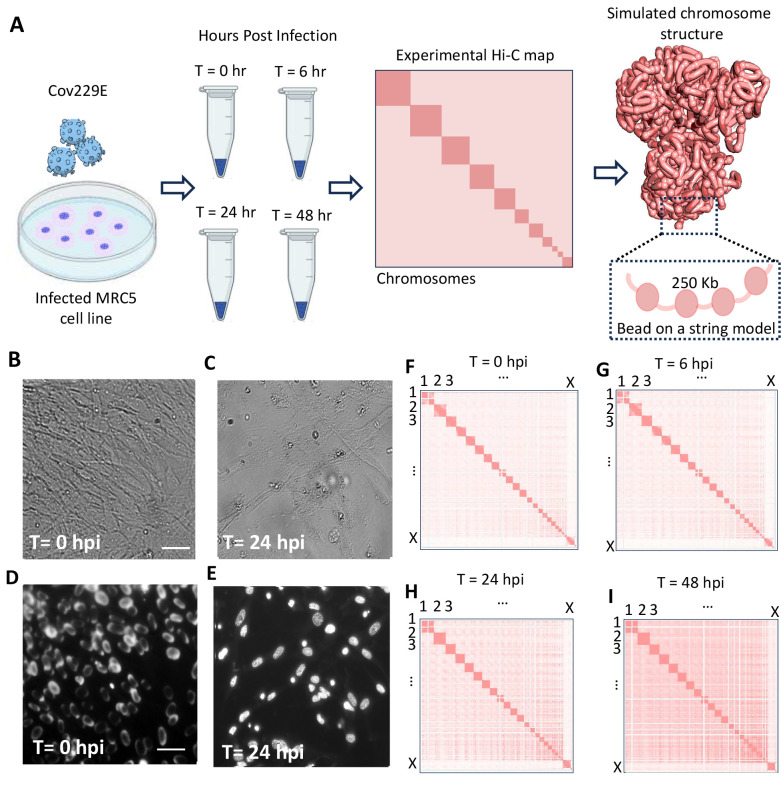
Common cold coronavirus (CoV229E) infection alters MRC5 cellular characteristics (A) Experimental data was generated via *α*-coronavirus 229E (CoV229E) infection of MRC5 cells. Samples were collected at four time points: 0-, 6-, 24-, and 48-hours post-infection for Hi-C. Sequencing data and contact maps were utilized for polymer modeling. (B-E) Image analysis of the MRC5 cell line infected with CoV229E. Cellular (top) and nuclear (bottom) morphology of MRC5 cells was examined using bright-field microscopy (B-C) and fluorescent imaging of DAPI-stained nuclei (D-E) to evaluate the impact of virus exposure. Representative images were chosen from each time point, and the scale bars correspond to 10 μm. Hi-C contact matrices were generated for (F) 0, (G) 6- (H) 24- (I) 48-hour post-infection time points acquired from each sample

We generated experimental data for analysis with 3D chromatin structural dynamics modeling. Samples from fetal lung fibroblasts (MRC5) cells infected with the *α*-coronavirus 229E strain (CoV229E) were collected at four time points, 0, 6, 24, and 48 hours post-infection (hpi) (Figure 1 A). Each sample was processed for in situ chromatin conformation capture and identification of long-range chromatin contacts. Cellular and nuclear morphology of MRC5 cells was examined throughout infection with CoV229E. Fluorescent images of DAPI- stained nuclei at 0-, 6- and 24- hours post-infection (hpi) indicate global chromatin alternations (Figure 1 B–E and Supplementary Figure 1). On average, 910 million read-pairs were sequenced across the libraries of four independent infection experiments to generate approximately 410 million unique contact pairs on average, per experimental condition (Supplementary Table 1). Simulated Hi-C contact maps were generated from experimental data to support modeling (see Figure 1 F–I).

**Figure 2:**
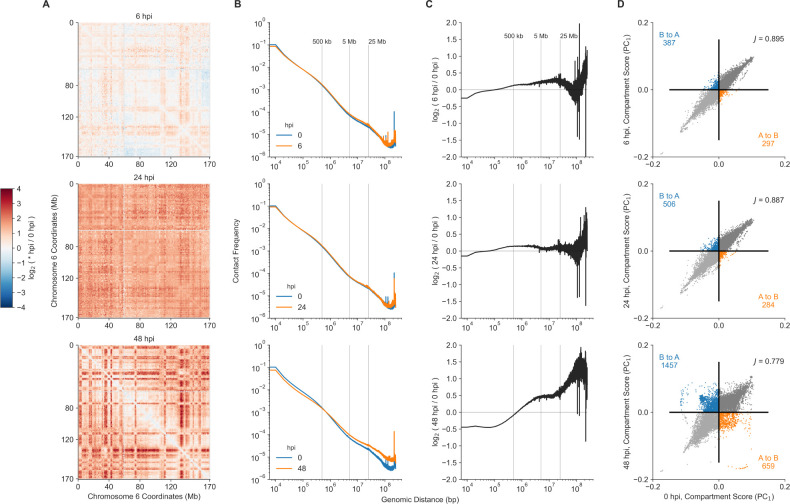
Hi-C analysis demonstrates the impact of *α*-CoV229E on the induced changes in MRC5 genome. (A) Fold change in Hi-C contacts every 1 Mb along chromosome 6 (x- and y-axis) at 6, 24, and 48 hpi (rows, top to bottom, respectively) compared against 0 hpi. Darker red and lighter blue colors indicate an increase or decrease in contact frequency (respectively) at either 6, 24, or 48 hpi compared to the 0 hpi Hi-C map. (B) Contact frequency (y-axis) along chromosomes as a function of genomic distance (x-axis) between the 0 hpi (blue) and CoV229E infected Hi-C experiments (orange) at 6, 24, and 48 hpi (rows, top to bottom, respectively). (C) The fold change of distance decay curves (shown in B) from Hi-C contacts at 6, 24, and 48 hpi (top to bottom, respectively) compared against the 0 hpi. (D) Compartment scores of Hi-C maps (at 250 kb resolution) at 6, 24, and 48 hpi (y-axis, top to bottom, respectively) plotted against the compartment scores of the Hi-C map at 0 hpi (x-axis). Grey colors depict compartment assignments consistent between the 0 hpi and the infection time points. Blue and orange dots mark 250 kb regions switching compartments from B to A or A to B (respectively). The Jaccard index (annotated in the upper right) measures the overlap of compartment assignments between the infected samples (6, 24, and 48 hpi) and the 0 hpi.

Within the Hi-C maps, we observed differences in contact frequencies among the time points for the Cov229E infection. Comparing the Hi-C map from cells collected at the initial infection timepoint (0 hpi), to the Hi-C map from samples collected at 6 hpi, there was a modest increase in the intensity of contacts, between distal regions, more than 500 kb away from each other (Figure 2 A, C). Similarly, there was a modest increase in contact intensity at 24 hpi (compared to 0 hpi), yet these differences were more uniform (for example along chromosome 6, Figure 2 A), with most of the differences in contact frequencies isolated to contiguous genomic regions less than 5 Mb apart (Figure 2 C). At 48 hpi, we observed the largest difference in the fold change of Hi-C contacts compared to contact frequencies calculated at 0 hpi (Figure 2 A). These included changes in the contact frequency of adjoining genomic regions and distant, intra-chromosomal contacts more than 500 kb apart (Figure 2 B, C). These changes in contact frequency within chromosomes are suggestive of alterations in genomic architecture due to Cov229E across time.

We also examined the change in chromosomal compartment assignments due to infection. For each chromosome, the A and B compartments of 250 kb regions were estimated by conducting principal component analysis and taking the value of the first principal component (PC1). Along a chromosome, 250 kb regions were listed as belonging to compartment A if the corresponding eigen value for that region was positive (PC1 > 0). Compartment scores were assigned via ATAC-seq data, which provides nucleosome positioning allowing for interpretation of open or closed regions of the genome. The open-ness of 250 kb regions (along each chromosome) was determined from ATAC-seq to properly orient open (A) and closed (B) compartments. The compartment scores (PC1) of genomic regions at 6, 24, and 48 hpi was compared to the compartment score at 0 hpi (Figure 2 D). We observed an increase in the switching of compartment assignments compared to the 0 hpi, with the greatest difference at 48 hpi. This switching of compartment assignments (off diagonal points in Figure 2 D) was quantified by a Jaccard index, which measures the overlap between the binary compartment assignments (A or B) at 0 hpi (Figure 2 D) with those at 6, 24, and 48 hpi (Figure 2D, top to bottom respectively). These results point to an alteration in compartment states—chromatin areas exchanged from A to B states—in the MRC5 genome due to Cov229E infection.

In addition to monitoring the dynamics of Hi-C contacts in the MRC5 genome, we also investigated changes in contact frequencies between chromosomes resulting from infection. Within each time point, the number of inter-chromosomal contacts (and the expected number of inter-chromosomal contacts) was counted between each pair of chromosomes using previously described methods [31]). From these counts, an interaction score was calculated [3] for every combination of chromosomes. With increased infection time, we observed stronger interaction scores between the smaller chromosomes (chromosomes 13 – 22 and Y) than between combinations of larger and smaller chromosomes (Supplementary Figure 2). Broadly, this pattern (which has been observed previously in other human cell-lines [32]) held across the different infection time points.

We then tested for strengthening and weakening of contacts between specific pairs of chromosomes in the Hi-C maps. For these tests, the contacts between chromosomes at 0 hpi were compared against the contacts between those same chromosomes at 6, 24, and 48 hpi, resulting in 828 comparisons between pairs of chromosomes. We detected an altered interaction frequency between chromosomes (compared to the 0 hpi) in approximately 65% of chromosome pairs (Fisher Exact Test, p-value < 1.21^−5^). Approximately 40% of the significant changes in inter-chromosomal contact frequency were observed between the 0 and 48 hpi time points (Supplementary Figure 3 A). Between the 0 and 6 hpi, significant increasing and decreasing of inter-chromosomal contacts were identified and distributed between pairs of smaller and larger chromosomes (Supplementary Figure 3 B). However, at 24 hpi the frequency of inter-chromosomal contacts between pairs of smaller chromosomes increased, while the contacts between pairs of larger chromosomes decreased, relative to the inter-chromosomal contacts at 0 hpi (Supplementary Figure 3 B). The pattern inverted at 48 hpi, with significantly stronger interaction between pairs of larger chromosomes, relative to 0 hpi (Supplementary Figure 3 B). Of the total 276 unique combinations of chromosome pairs, approximately 36% of pairs (on average) had no significant alteration in contact frequency (neither increase nor decrease) at the time points when compared to 0 hpi (Supplementary Table 2).

### 4D Hi-C polymer modeling of chromatin dynamics induced by CoV229E infection

We used the 4D-HiC method [20] to model how CoV229E induces changes in individual chromosomes in the MRC5 genome. We utilized the polymer bead-spring model, which is a widely used method for studying genomic organization [33, 34]. We incorporated a harmonic constraint in the form of pairwise interaction extracted from experimental Hi-C contact maps to simulate the crosslinking contacts in the polymer. This allowed us to achieve a 3D model that was experimentally validated and corresponded to Hi-C maps. It is important to note that our approach made no assumptions about chromosome structures and was solely based on experimental Hi-C maps. We used a resolution of 250 kb for all the chromosomes. For example, chromosome 1 (249 Mb) was represented by a polymer with 996 coarse-grained (CG) beads, where one CG bead represents each Hi-C bin at 250 kb resolution. Although this resolution loses fine structural details such as loops, it still allows us to identify global genomic features, including compartments. All simulations began with a random polymer chain conformation, resulting in a 3D organized polymer based on experimentally extracted harmonic constraints. To achieve convergence, simulations were iteratively compared between the experimental Hi-C maps and simulated 2D contact maps by analyzing the correlation coefficient (>0.75). The number of CG beads in our model can be increased or decreased based on the resolution of the experimental Hi-C dataset.

**Figure 3:**
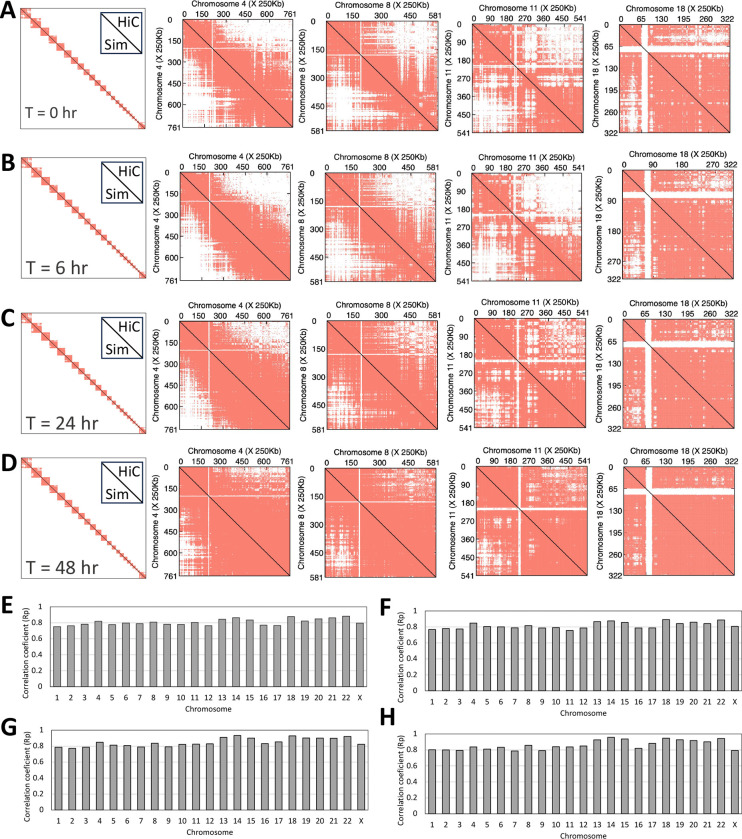
Polymer simulations succeed in reproducing intra-chromosomal contacts measured in Hi-C experiments. Comparison between experimental (upper diagonal) vs. simulated (lower diagonal) Hi-C contact maps for the time points (A) 0 hpi, (B) 6 hpi, (C) 24 hpi, and (D) 48 hpi with individual Hi-C maps for chromosomes (i) 4, (ii) 8, (iii) 11, and (iv) 18 shows agreement with Rp> 0.80 in all cases. Correlation coefficients between simulated and experimental intra-chromosomal contacts for each chromosome are shown for time points (E) 0 hpi, (F) 6 hpi , (G) 24 hpi, and (H) 48 hpi.

The initial models generated by implementing 2D Hi-C contacts resulted in a folded chromosome model for each chromosome. Each simulated Hi-C map for each chromosome was compared against the experimentally derived Hi-C contact maps (Figure 3) for every time point (Supplementary Figures 4–7). Specific comparisons of chromosomes 4, 8, 11, and 18, which show the greatest correlations but also the distinctive variation in contact frequencies among different chromosomes, are shown as an inset in Figure 3(A–D). These data provide insight into visualizing the 3D complex features observed from a 2D experimental contact map with an added time dimension. The simulated 2D contact maps correlate strongly with the experimental Hi-C maps, with point-to-point Pearson correlation coefficients ranging from 0.75 to 0.95 (Figure 3 E–H). The simulated Hi-C maps have successfully recapitulated similar changes as experimental Hi-C, with a combined Pearson correlation coefficient of 0.80 when averaged over all chromosomes. We also observed an enhancement in intra-chromosomal and long-distance interactions in our simulated Hi-C maps, which is consistent with the empirical findings. However, it is important to note that experimental Hi-C contact maps contain unmappable regions, which are assumed to be the centromeric regions in the chromosome. It is not possible to know the exact connectivity of these specific segments based on our approach. Given there are no specified Hi-C harmonic constraints for this area, the unmappable regions were excluded from further analysis. Nonetheless, it is important to emphasize that only experimental Hi-C map data is used to generate 3D models, and the agreement between the simulated and experimental Hi-C provides sufficient support to perform further structural analysis.

### Quantification of changes in A/B compartmentalization in the MRC genome induced by CoV229E infection

**Figure 4:**
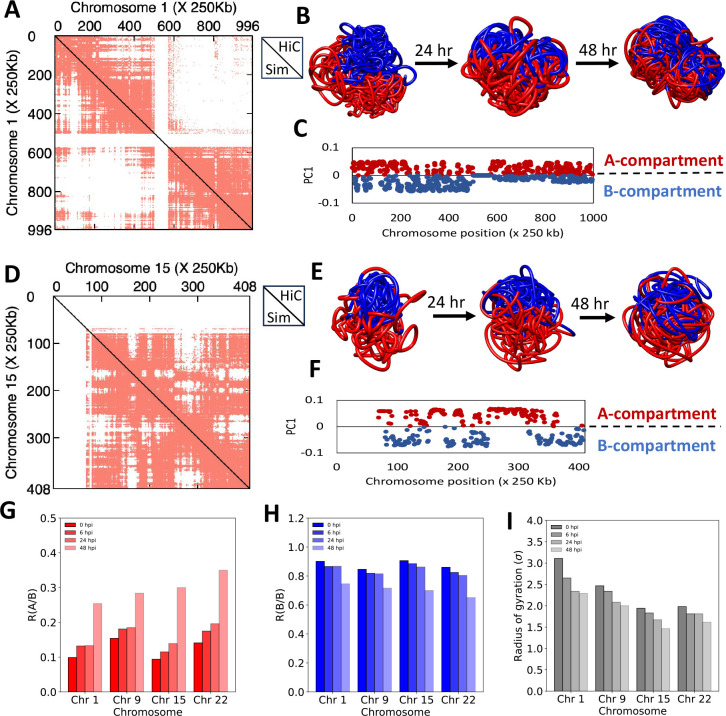
Quantification of mixing of A/B compartments in response to CoV229E infection using polymer modelling. (A) Hi-C contact profiles for chromosome 1 between the experimental 0 hpi time point Hi-C map (lower diagonal) and simulated Hi-C map (upper diagonal). The point-to-point Pearson correlation coefficient is Rp = 0.76. (B) A/B compartments defined by PC1 of chromosome 1 projected onto 3D reconstruction, where red indicates positive PC1 values and blue indicates negative PC1 values. ((C) Genome-wide PC1 distribution for chromosome 1. (D) Hi-C contact profiles for chromosome 15 between the experimental 0 hpi time point Hi-C map (lower diagonal) and simulated Hi-C map (upper diagonal). The point-to-point Pearson correlation coefficient is Rp = 0.84. (E) A/B compartments defined by PC1 of chromosome 15 projected onto 3D reconstruction. (F) Genome-wide PC1 distribution for chromosome 15. (G) To quantify mixing within the compartment A and B, the ratio RA/B for chromosomes 1, 9, 15, and 22 shows an increase during viral infection. (H) For comparison, a similar ratio is computed for interactions between B compartment beads (RB/B). As expected, the number of these interactions decreased due to mixing with the A compartment. (I) Comparison between the radius of gyration (Rg) of individual chromosomes is shown for all the time points post-infection.

The PC analysis of experimental Hi-C maps indicates compartment changes upon viral infection. While the calculated PC values provide valuable insights, they are still 1D in nature. Understanding the spatial organization of these compartments in 3D can provide more structural insights. To achieve this, the A and B compartment scores were mapped onto simulated 3D models. We observed that A/B compartments were spatially segregated in 3D for each chromosome at the 0 hpi timepoint (Figure 4 A, B). Additionally, the B compartment was more compact than the A compartment, which is consistent with their structural characteristics. To visually investigate the compartment mixing, we mapped the A and B compartment data from the 0 hpi to the other time points. We observed systematic mixing of the compartments across the time points. There was a slight increase in compartment mixing at 0, 6, and 24 hpi, along with the overall compaction of both compartments. However, a more dramatic increase in A-B mixing was observed at 48-hpi compared to earlier time points (Figure 4 B, E).

To quantify the mixing of A and B compartments over time, we computed a mixing ratio R(A/B). The number of interactions between A and B compartment beads were calculated and divided by the total number of interactions made by beads for each compartment. The ratio was determined for chromosomes 1, 9, 15, and 22, where originally well-separated A-A/B-B interactions were observed at the initial 0 hpi time point; these interactions were altered after CoV229E infection at 6, 24, 48 hpi, and A-B interactions were enhanced (Figure 4 G, H). The R(A/B) increased drastically for all the considered chromosomes at 48 hpi, while the R(B/B) ratio decreased with the rate of infection due to compartment B mixing with compartment A (Figure 4 G, H). However, the rate of mixing varied across different chromosomes. For example, chromosome 1 showed no difference between 6- and 24- hpi, while chromosomes 15 and 22 showed an increase in R(A/B) mixing ratio between 6- to 24-hpi. Potentially, intra-B interactions were replaced by interactions between A/B compartments due to viral infection, where the increased R(A/B) ratio indicates weakened A and a closed chromatin state, similar to B compartment features.

During the calculation of compartment mixing, we observed that viral infection caused chromosome compaction. To measure the degree of compaction, we calculated the radius of gyration (Rg) for all the chromosomes under consideration. The Rg value decreased over time for all chromosomes, indicating an increase in chromosome compactness due to viral infection. However, the degree of compaction varied across chromosomes and time points. Most chromosomes showed only a slight decrease in Rg from their initial state at 6 hpi (Figure 4 I). For instance, chromosome 4 showed negligible change from 0 to 6 hpi, but almost a 25% reduction in Rg at 48 hpi compared to its initial state.

### Prediction of CoV229E impacts on gene structuring

**Figure 5:**
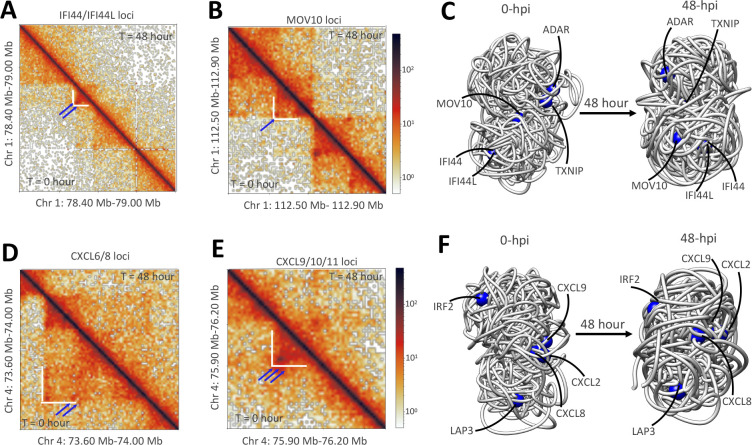
Global structural changes in the pro-inflammatory and Interferon gamma genes positions post-infection. **(A-B)** Experimental Hi-C maps (bin size, 5Kb) for chromosome 1 between t = 0 hour (lower diagonal) and t = 48 hour (upper diagonal). Here, the blue arrow denotes the reduced dot-shaped loops, and the white line represents the surrounding area. **(C)** Three-dimensional reconstruction of chromosome 1 with genes represented as blue spheres. **(D-E)** Experimental Hi-C maps (bin size, 5Kb) for chromosome 4 between t = 0 hour (lower diagonal) and t = 48 hour (upper diagonal). **(F)** Three-dimensional reconstruction of chromosome 4 with genes represented as blue spheres.

Studies have previously shown that viral infection induces two immuno-pathological features: an increase in pro-inflammatory cytokines and a weakened or decreased immune response [35]. For example, SARS-CoV-2 infection causes activation of pro-inflammatory genes (PIF) but reduced activation of interferon-stimulated genes (ISGs) [12, 35]. To understand the impacts of less pathogenic viruses, we analyzed changes in gene 3D spatial position due to CoV229E. The 0 hpi time point (initial stage) was compared to the 48 hpi time point (later stage). We found changes in chromatin structuring, as loops were diminished in the vicinity of gene loci. The intra-chromosome contacts also decreased significantly in the region for chromosome 1 (Figure 5 A,B). We further examined the region containing the CXCL family gene loci on chromosome 4 and found a similar phenomenon (Figure 5 D, E).

To examine the spatial distribution of genes, we mapped them onto our simulated models and observed whether they were located in the core, periphery, or on the surface of the chromosomes. This allowed us to visualize and comprehend gene organization changes in 3D. Previous work has shown that silent genes tend to localize within the core of the chromosome, while active genes are more likely to be found on or beneath the surface of the X-chromosome [36, 37]. We observed that the chromatin region containing ISGs (indicated by the blue sphere) on chromosome 1 was located near the surface at 0 hpi. This region relocated to the core of the chromosome after CoV229E infection at 48 hpi (Figure 5 C). Similarly, the chromatin region containing CXCL family genes (PIF) on chromosome 4 was located just beneath the surface of the chromosome (Figure 5 F) at 0 hpi; yet, this region remained on the surface at 48 hpi. These findings suggest that the region containing the IFN genes of chromosome 1 becomes buried in the chromosome core in response to CoV229E infection, while regions containing PIF genes in chromosome 4 prefer to reside at the chromosome surface. Our model offers a method for visualizing regions containing specific genes and their restructuring in 3D and illuminates regions that should be further investigated for their biological significance.

### Effect of CoV229E on X chromosome

**Figure 6:**
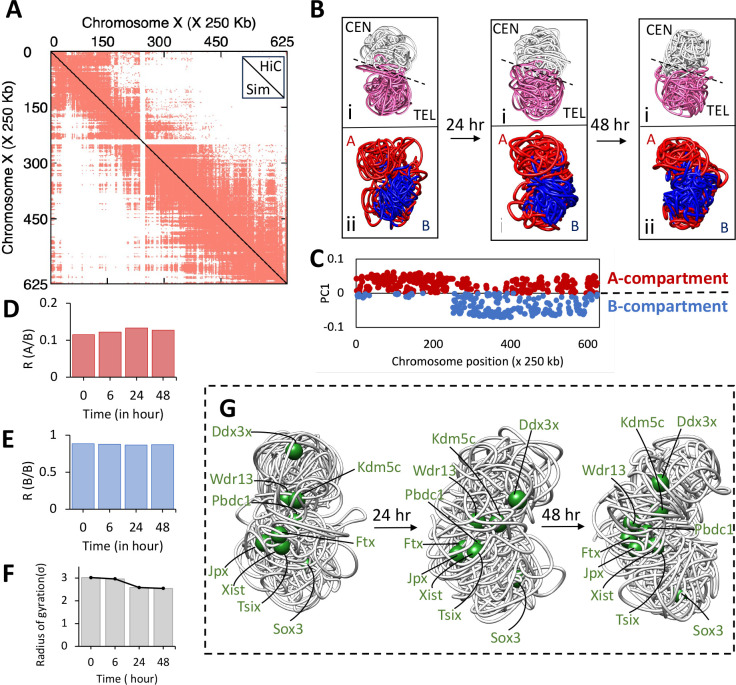
*α*-CoV229E effect on male X-chromosome. Hi-C contact profiles for chromosome X between the experimental Hi-C map at 0- hpi (lower diagonal) and simulated Hi-C map at 0- hpi (upper diagonal). The point-to-point Pearson correlation coefficient is Rp = 0.80. (B) Results show the mixing of proximal and distal regions. Pink corresponds to proximal halves (positions 0 to 73 Mb); white corresponds to the distal region (positions 73 to 166 Mb). A/B compartments defined by PC1 for chromosome X projected onto 3D reconstruction show spatial separation between A and B compartments. Red: positive values in PC1; blue: negative values with respect to time as (i) t = 0 hr, (ii) t = 24 hr, and (iii) t = 48 hr. (E) Genome-wide PC1 distribution for chromosome X at t = 0 hr. (F) To quantify mixing within the compartment A and B, the ratio RA/B was computed for chromosome X and showed no change during viral infection. (G) For comparison, a similar ratio is computed for interactions between B compartment beads (RB/B). (H) Comparison between the radius of gyration (Rg) for X chromosomes for all the time points post-infection.(I) Three-dimensional reconstruction of chromosome X with escapee genes represented as green spheres. Escapee genes remain localized on the chromosome surface, implying no effect of CoV229E on the chromosome.

While viral infection can lead to widespread changes in genomic architecture, there were only minor structural changes found in the X-chromosome. The MRC5 is a male cell line with only one X chromosome; previously, it has been demonstrated that the sex chromosomes behave differently. For example, X chromosome-linked gene called UTX was found to act as an epigenetic regulator to boost NK cell anti-viral function and to repress NK cell numbers [38]. Similarly, ACE2 gene, encoding for angiotensin-converting enzyme 2 and primary receptor for SARS-CoV infection, is localized on the X chromosome and was identified as an ISG. To understand the impact of CoV229E on X chromosome, we calculated contact frequency (Supplementary Figure 8). Contact frequency analysis revealed that only small changes occurred at 6 hpi in the X chromosomes, with almost no change observed at 24 and 48 hpi. Our simulations of chromosomes found increased compactness and inter-mixing of the A/B compartment for all chromosomes in response to CoV229E infection. Therefore, we modeled the X chromosome following the same protocol for all four time points post-infection. The simulated Hi-C contact map strongly correlates with the experimental Hi-C map for all-time points post-infection (Figure 6A and Figures S3-S4).

The X chromosome is divided into two halves, namely proximal and distal, separated by a micro-satellite repeat called Dxz4. The proximal half spans from 0 to 72 Mb, while the distal half corresponds to 73 to 166 Mb region [39, 40]. These regions were mapped onto 3D models, with the proximal half represented in white and the distal half in pink (see Figure 6 B). Previous studies have shown that the two halves form compact globular structures that are spatially separated in the 3D model. Interestingly, no changes were observed in the mega-domain structure during viral infection, and they remained intact up to 48 hours post-infection. A PC analysis of Hi-C data on the X chromosome was also performed, and the 1D data was mapped onto the simulated 3D models. However, no mixing of A (red) and B (blue) compartments was observed in the compartments during the latter stages of viral infection (refer to Figure 6 B). The R(A/B) and R(B/B) ratios were calculated to quantify the mixing, and the R(A/B) ratio only slightly increased at 6- and 24- hpi. Surprisingly, the R(A/B) ratio reduced slightly at 48-hpi, suggesting a compensatory response to revert back to its initial state (Figure 6 D, E). The radius of gyration (Rg) was also calculated, and slight compaction was observed between 6 hpi and 24-hpi; this level of compaction was maintained in the 48 hpi time point (Figure 6 F). This compaction can be attributed to the overall compaction of the whole genome.

To ensure the effect of CoV229E on X-chromosomes is not due to sampling difference because of only a single X chromosome. We randomly removed half of the valid Hi-C contacts mapping to chromosomes 1 through 22, leaving chromosomes X and Y. We followed the same protocol to model chromosomes 1, 9, 15, and 22 at 0- and 48- hpi to study the effect of Hi-C map down-sampling. Despite the reduction in Hi-C contact, we still observed a strong correlation between the down-sampled experimental Hi-C and simulated Hi-C for both the initial and later stages of infection (see Supplementary Figure 9), which provided further evidence of the capability of the developed method. The radius of gyration was calculated and compared with the original Hi-C maps. We observe a slight increment in the Rg for most of the cases. This could be attributed to the reduced number of Hi-C contacts. However, comparing the change % of Rg with the original Hi-C maps showed a very slight difference. Overall, the down-sampled Hi-C-based simulated model displays similar compaction as the original Hi-C maps (see Supplementary Figure 9). We made a similar observation while calculating the R(A/B) and R(B/B) for the down-sampled models. Although a slightly reduced mixing was observed with down-sampling, the overall trend correlated with the original Hi-C maps (see Supplementary Figure 9). These results suggest that the CoV229E has a significantly reduced effect on the male X-chromosome compared to other chromosomes, irrespective of the Hi-C contact read depth difference.

We also investigated whether viral infection induces any changes in the gene structuring of X chromosomes. We specifically focused on genes that are known to be involved in the transition from active to inactive states of the X-chromosome (see Figure 6 G). These were mapped on the simulated 3D X chromosome model. In females, these genes cluster together, ranging from 100 kb to 7 Mb [41, 42] and are predominantly found in pseudoautosomal regions (PAR) [43]. Using the same procedure, we mapped the chromatin region containing these genes at 0-, 24-, and 48-hpi. Consistent with previous work, this chromatin region resided near the chromosome surface [36, 37]. Furthermore, the genes of interest were found in small clusters (Figure 6 G); however, there was no significant gene loci displacement at 24- and 48-hpi, suggesting that CoV229E does not affect the X chromosome in males.

## Discussion

Understanding the impact of viruses on genomic architecture has become increasingly important in light of the recent pandemic. It is crucial to understand the changes that occur in host chromatin due to viral infection as a means to develop anti-viral strategies and countermeasures. Recent advancements in experimental techniques, including Hi-C and ATAC-seq, along with other related methods, have revealed specific chromosomal features, including CTCF binding sites, TADs, chromatin loops, and transcriptional compartments. By combining these high-throughput sequencing techniques with different modeling techniques, we can gain a better understanding of genome organization [20, 44, 45].

In this study, we used a hybrid approach to investigate the restructuring of 3D chromatin by a common-cold coronavirus called *α*-CoV229E. By analyzing experimental Hi-C maps, we observed significant changes in the genomic architecture due to viral infection. We found that the host chromatin exhibited changes at various time points post infection. Our analysis also revealed an oscillatory behavior in the contact frequency as a function of genomic distance, reflecting the dynamic nature of the genome with viral infection. Previous studies on SARS-CoV-2-infected A549 cell lines have shown a loss of intra-TAD chromatin contacts, while mild changes were reported for the common-cold coronavirus HCoV-OC43 strain [12]. Similar to SARS-CoV-2, it could also be deduced that CoV229E might also deplete the cohesin complex post-infection [12], which explains the long-range association among the chromosomes. However, more functional and comparative data will be needed to support these findings. At 48 hpi, CoV229E showed a slightly similar effect as SARS-CoV-2 at 24 hpi, which can be attributed to the lower infectious rate of CoV229E. We also observed widespread changes in A/B compartment structures, with more B-to-A and reduced A-to-B switching occurring with viral infection. This is in line with the 30% of genomic compartmental weakening or switching found in response to SARS-CoV-2 infection [12].

Here, we have also presented polymer models that can help study the structural changes that occur due to viral infection. Our approach (4DHiC) is based on experimental data, where we derived harmonic constraints from experiments. We observed a strong correlation between the 3D models and the experimental results, which is interesting considering the simplicity of our method. The method also worked quite incredibly with the down-sampled experimental Hi-C contacts maps, further demonstrating the capability of the method. Importantly, the 3D models also allowed us to quantify changes and details that were not discernible from the experimental Hi-C maps. We projected the experimental data onto a 3D model and examined the compartment mixing. We also calculated the ratio of compartment mixing with respect to time post-infection and found a significant increase in A/B mixing in response to infection, which is consistent with our experimental findings. Our 3D models also revealed that chromosome compactness increases with viral infection. This increased compactness leads to further interaction within the chromosome, indicating increased intra-chromosomal contacts. We inferred that the chromosomes become more densely packed within the chromosome’s core upon viral infection and are likely transitioning to the heterochromatin state, though the direct link between chromatin compaction and gene expression requires more empirical data.

Our simulation also revealed that changes in genomic architecture induced by CoV229E also induced relocation of regions containing ISGs and PIF gene loci. Regions containing IFN genes in chromosome 1 localized within the core of the chromosome during viral infection. In contrast, regions containing PIF genes in chromosome 4 resided on or just below the chromosome’s surface with no change in location in response to infection. This concurs with a similar observation of SARS-CoV-2 infection in the A549 cell line, which is not particularly permissive compared to the MRC5 cell line. Our simulation demonstrates that the PIF and IFN genes play a critical role in viral infection and could be a point of interest for developing therapeutics for patients suffering from *α*-Cov229E infection.

Recent research has focused on the X-chromosome to better understand the molecular factors responsible for sex differences in antiviral immune responses. It is speculated that viral infections could alter the 3D architecture of the X chromosome. Here, we found that CoV229E infection within MRC5 cells causes limited changes in the structure of the X-chromosome. Subsequently, our simulation also revealed no significant compartment mixing or genomic restructuring occurred due to viral infection in the X chromosome. We also tested the down-sampled Hi-C maps by removing half of the valid Hi-C contact and found the changes persisted over the other chromosomes. This provides sufficient evidence that CoV229E behaves differently with the X-chromosome. These findings also suggest that more studies are needed in this direction to fully understand the relationship between CoV229E and the X-chromosome.

Collectively, our simulation found that Cov229E is capable of inducing significant structural changes in the genomic architecture of MRC5 cells that are highly permissive to virus replication. Our simulations also suggest that an endemic, less virulent coronavirus alters host chromatin architecture, providing deeper insights into outcomes of non-pathogenic viruses on host genomic structures. Finally, we demonstrate that our model is transferable across chromosomes and provides more insights when used in synergy with experimental data.

## Methods

### Cell culture and virus infection experiments

Human fetal lung fibroblast MRC5 (ATCC, CCL-171) were maintained in DMEM (Gibco, ThermoFisher Scientific) or EMEM (Gibco, ThermoFisher Scientific) media, respectively, supplemented with 10% FBS (Hyclone). Human coronavirus 229E was obtained from ATCC (VR-740) and was propagated in MRC5 cells. Concentrated virus stocks were prepared by pelleting virus particles from 0.2 *μ*m syringe filters) conditioned EMEM media via centrifugation for 18 h at 50,000g. To calculate the concertation of infectious virus particles, virus pellets were resus- pended in serum free EMEM and were titrated on MRC5 cultures that were 90% confluent. Virus stocks were serially diluted in EMEM containing 2% FBS. Diluted virus was incubated with MRC5 cultures at 35°C for 5 days and virus titer was determined based on visual cytopathic effect (CPE) using bright field and fluorescent microscopy (Live/Dead assay, ThermoFisher Scientific, L3224). The infectious dose at 50% (TCID50) per milliliter infectious dose was determined from the highest dilution of virus stock at which 50% of the cells showed CPE applying the Reed-Muench method [46]. For viral infection experiments, MRC5 cells were plated in four separate 10 cm culture dishes and were exposed to virus at 100% confluency (to avoid variability in spatial chromatin dynamics due to cell division). Virus concentration at multiplicity of infection (MOI) of 1 was added to each culture at 1 ml volume (to optimize the absorption phase). Infected cells were incubated at 35 °C, with 5% CO2 for 1 h, followed by addition of 8 ml fresh 2% EMEM media and continued incubation for additional time until cell harvesting for each culture at 5 h, 24 h, and 48 h post infection. Cells were collected for Hi-C and ATAC-seq analysis.

### Hi-C protocol for in situ chromatin conformation capture

Cells were harvested, resuspended in culture media, counted, and adjusted to 10^6^ per milliliter. The four experimental samples were processed following Arima-HiC Kit User Guide Mammalian Cell Lines protocol (ARIMA Genomics, A510008). Briefly, cells were cross- linked with methanol-free formaldehyde (final concentration 2%) for 10 min at RT with continuous mixing. Stop solution #1 was added for 5 min at RT followed by 10 min incubation on ice. Cells were pelleted by centrifugation, washed with PBS, and distributed in 106 cell aliquots. Cell pellets were stored at −80°C until further processing. Cell lysis, enzymatic DNA digestion, fill ends, biotin ligation, and reverse crosslinking were performed according to the manufacturer’s manual. DNA was purified with AMPure XP beads and sheared to 400 bp fragments using the Covaris Focused-Ultrasonicator instrument. On-beads libraries were prepared for Illumina NextSeq2000 with the NEBNext Ultra II DNA Library Prep kit (NEB, E7645L) following the manufacturer’s protocol. Library preparation and sequencing was performed at the Los Alamos Genome Center.

### Hi-C Map Generation and Analysis

Fastq.gz files from paired end Illumina sequencing were analyzed using the Juicer Hi-C pipeline [47]. These samples were aligned to the GRCh38 homo sapiens reference genome from the ENCODE consortium [48]. Prior to alignment, the reference fasta file was filtered to only include the twenty-two largest contigs (representing the autosomes), the X and Y chromosomes, and the mitochondrial contig. Restriction sites associated with the Arima protocol were identified using tools within the juicer pipeline. Hi-C files (.hic) were constructed using default settings within juicer, and for subsequent analysis the quality threshold of ‘q = 30’ was used.

Technical replicate Hi-C maps were merged using the “mega” function in the juicer pipeline. The number of sequenced read pairs of these merged Hi-C maps ranged from 839.9 million to 1.402 billion, with a median of approximately 910.2 million sequenced read pairs (Supplementary Table 1). The number of Hi-C contacts across samples, after alignment and filtering, ranged from 387.2 to 629.6 million, with a median of approximately 409.7 million, across combined libraries generated here (Supplementary Table 2).

To monitor the distance decay of Hi-C contacts, the “expected” function in FAN-C [49] was used to calculate frequencies at a resolution of 10 kb for each Hi-C map across the infection time course. The A and B compartment scores were calculated using the “eigenvector” function in juicer tools.

### ATAC-seq profiling for nucleosome locations

As previously described, ATAC-seq libraries were generated from live-cell harvesting of cells at 0, 6, and 24 hr post infection [50]. Approximately, 80,000 cells from each sample were processed using the Active Motif ATAC-seq kit (Cat #53150) per the manufacturer’s instructions. Briefly, cells were washed, lysed in buffer provided, and centrifuged at 4°C for 10 min. DNA was tagmented, purified, and libraries were generated using the indexed primers provided. AmPureXP beads were used for further size section to remove unneeded fragments. Libraries were quantified and sequenced using the Illumina NextSeq2000 in PE151 (paired end) mode with P3 chemistry. Libraries were sequenced at the Los Alamos Genome Center.

### ATAC-seq alignment and filtering

ATAC-seq paired reads were analyzed using previously described methods [50]. Briefly, reads were trimmed to remove Nextra adaptors and then filtered to remove reads with repetitive sequences and low quality scores (q < 15) via Fastp [51]. These processed reads were aligned to the GRCh38 homo sapiens reference genome from the ENCODE consortium [48] via bwa [51]. Read pairs mapping to the mitochondria or those marked as duplicates (via samblaster [52]) were removed from further consideration.

### Peak calling of ATAC-seq data

Filtered ATAC-seq alignments were further analyzed to identify peaks formed by the concentrated mapping(s) of paired-end reads. These loci were identified via the peak caller, MACS2 [53]. Input samples were filtered using the samtools [54] view command (and the following samtools flags: -F 4 -F 256 -F 512 -F 1024 -F 2048 -q 30) and then passed to MACS2, using the bampe setting. In MACS2, the min-length and max-gap settings of 100 and 150 bp (respectively) were used. The calling summits option was also used.

Along each chromosome, the number of peaks every 250 kb were counted. These counts were correlated with estimates of Hi-C compartments calculated via juicer tools [47]. Here it was assumed bins with higher counts of peaks are considered more open, and should belong to compartment A. Where needed, the eigen values of Hi-C compartment scores or an entire chromosome were multiplied by negative one to correctly orient open and closed compartment assignments across the genome.

### Inter-chromosomal Analyzes

For each time-point post infection, contacts between chromosomes were counted every 250 kb from Hi-C files using hicstraw [55]. Frequencies of inter-chromosomal interactions within Hi-C maps were calculated using an interaction score (as seen in [31]) and visualized using the Seaborn package in python [56]. To compare inter-chromosomal contacts at different time points after 229E infection, a Fishers exact test was used to identify changes in the total inter-chromosomal interactions, comparing the counts of Hi-C contacts between chromosomes to the total number of inter-chromosomal contacts (as described in [57]). At the time points, this procedure resulted in 828 unique tests, each comparing the interaction frequency between a pair of chromosomes at 6, 24, and 48 hpi to their interaction at 0 hpi. To adjust for multiple testing, a Bonforoni correction was applied, adjusting the cutoff of significance from an alpha = 0.01 to an alpha ~ 0.01/828 < 1.20^−5^.

### Computational setup

All the simulations were performed with the Large-Scale Atomic/Molecular Massively Parallel Simulator (LAMMPS) [58]. Each chromosome is composed of several coarse-grained(CG) beads where each bead corresponds to ≈ 250 Kb, and the overall number of CG beads depends upon the chromosome length. The initial structure as a connected chain was generated using a self-avoiding random walk algorithm. The pair interaction between each CG bead was modelled using FENE and Lennard-Jones potential.

(1) $$U_{FENE-LJ}=-\frac{1}{2}\kappa R_{o}^{2}log\left[1-{\left(\frac{r}{R_{o}}\right)}^{2}\right]+\left\{\begin{array}{ll} 4\epsilon \left[{\left(\sigma /r\right)}^{12}-{\left(\sigma /r\right)}^{6}+\epsilon \right], & r\leq 2^{1/6}\sigma \\ 0, & r>2^{1/6}\sigma \end{array}\right.,$$

Here, $R_{o}$ is the parameter for the maximum bond length =20σ where σ correspond to distance in LJ units and the spring constant κ is $30\;\epsilon /\sigma^{2}$. For non-bonded interaction between atoms, repulsive Lennard-Jones interaction potential was used:

(2) $$U_{LJ}=\left\{\begin{array}{ll} 4\epsilon \left[(\sigma /r{)}^{12}-(\sigma /r{)}^{6}+\frac{1}{4}\right], & r\leq r_{o}\\ 0, & r>r_{o} \end{array},\right.$$

Experimental Hi-C constraint were implemented as harmonic constraints to simulate the cross-linking contact. To avoid noise from low intensity signals, the frequency of contact of Hi-C data was set to be greater than 10. The simulation temperature was kept constant using the Langevin thermostat at 1 in reduced units with damping coefficient was set to $1\tau^{-1}$. A timestep of 0.01τ was used, where τ is the reduced (Lennard-Jones) time. The polymer with harmonic constraints were subjected to Brownian dynamics with implicit solvent condition. To test the similarities between the experimental and simulated Hi-C map, Pearson correlation coefficient was calculated. The in-built LAMMPS routines were used to calculate the radius of gyration (Rg).

## Data Availability

The raw paired-end fastq files from experiments described here (both ATAC-seq and Hi-C) are published under the bioproject PRJNA975595 with accession numbers SRR27878167 – SRR27878173.
